# Prevalence, risk factors and molecular epidemiology of highly resistant gram negative rods in hospitalized patients in the Dutch region Kennemerland

**DOI:** 10.1186/s13756-016-0107-6

**Published:** 2016-03-08

**Authors:** Dennis Souverein, Sjoerd M. Euser, Bjorn L. Herpers, Bram Diederen, Patricia Houtman, Marina van Seventer, Ingeborg van Ess, Jan Kluytmans, John W. A. Rossen, Jeroen W. Den Boer

**Affiliations:** Department of Epidemiology and Infection Prevention, Regional Public Health Laboratory Kennemerland, Boerhaavelaan 26, 2035 RC Haarlem, The Netherlands; Department of Infection Prevention, Spaarne Gasthuis, Haarlem, The Netherlands; Department of Infection Prevention, Rode Kruis Ziekenhuis, Beverwijk, The Netherlands; Laboratory for Microbiology and Infection Control, Amphia Hospital, Breda, and University Medical Center, Utrecht, The Netherlands; Department of Medical Microbiology, University Medical Center Groningen, University of Groningen, Groningen, The Netherlands

**Keywords:** HR-GNRs, ESBL, Prevalence, Risk factors, Whole genome sequencing, Netherlands

## Abstract

**Background:**

This paper describes (1) the Highly Resistant Gram Negative Rod (HR-GNR) prevalence rate, (2) their genotypes, acquired resistance genes and (3) associated risk factors of HR-GNR colonization among the hospitalized population in the Dutch region Kennemerland.

**Methods:**

Between 1 October 2013 and 31 March 2014, cross-sectional prevalence measurements were performed in three regional hospitals as part of each hospitals infection control program. Rectal swabs were analyzed at the Regional Public Health Laboratory Kennemerland by direct culturing. Genotypes and acquired resistance genes of positive isolates were determined using Whole Genome Sequencing with the MiSeq instrument (Illumina). Association between several independent variables and HR-GNR positivity was examined using logistic regression models.

**Results:**

Out of 427 patients, 24 HR-GNR positive isolates were recovered from 22 patients, resulting in a regional HR-GNR colonization prevalence (95 % CI) of 5.2 % (3.6–7.9). Of these 22 positive patients, 15 were Extended Spectrum Beta-Lactamase (ESBL) positive (3.5 % (2.1–5.7)), 7 patients were positive for a Fluoroquinolones and Aminoglycosides (Q&A) resistant Enterobacteriaceae (1.6 % (0.8–3.3)) and from one patient (0.2 % (0–1.3)) a *Stenotrophomonas maltophilia* resistant towards co-trimoxazole was isolated. No carbapenemase producing Enterobacteriaceae (CPE), multi-resistant *Acinetobacter* species or multi-resistant *Pseudomonas aeruginosa* were isolated. The ESBL genes found were *bla*_CTX-M-1_ (*n* = 4, 25.0 %), *bla*_CTX-M-15_ (*n* = 3, 18.8 %), *bla*_CTX-M-27_ (*n* = 2, 12.5 %), *bla*_CTX-M-14b_ (*n* = 2, 12.5 %), *bla*_CTX-M-9_ (*n* = 2, 12.5 %), *bla*_CTX-M-14_ (*n* = 1, 6.3 %), *bla*_CTX-M-3_ (*n* = 1, 6.3 %), *bla*_SHV-11_ (*n* = 1, 6.3 %) and *bla*_SHV-12_ (*n* = 1, 6.3 %). Being known HR-GNR positive in the past was the only significant associated risk factor for HR-GNR positivity, odds ratio (95 % CI): 7.32 (1.82–29.35), *p*-value = 0.005.

**Conclusions:**

Similar ESBL prevalence rates and genotypes (3.5 %) were found in comparison to other Dutch studies. When previously HR-GNR positive patients are readmitted, they should be screened for HR-GNR colonization since colonization with GR-GNRs could be prolonged. We recommend for future studies to include all defined HR-GNRs in addition to ESBLs in prevalence studies, in order to obtain a more comprehensive overview of colonization with HR-GNRs.

## Background

Worldwide there is an alarming increase in the prevalence of Highly Resistant Gram Negative Rods (HR-GNRs) among clinical isolates [1–3]. The emergence and spread of HR-GNRs is a public health threat since infections caused by HR-GNRs are associated with an increased risk of morbidity, mortality, and healthcare costs (estimated mean additional costs per case between € 5449.- and € 27,245.-) compared to susceptible micro-organisms [4, 5]. In the Netherlands, the group of HR-GNRs is defined as (1) Enterobacteriaceae that are Extended Spectrum Beta-Lactamase (ESBL) and/or carbapenemase positive (CPE) and/or resistant towards Fluoroquinolones and Aminoglycosides (Q&A), (2) *Acinetobacter* species that are CPE and/or resistant to Q&A, (3) *Stenotrophomonas maltophilia* resistant towards co-trimoxazole and (4) multi-resistant *Pseudomonas aeruginosa* (Table 1) [6].

**Table 1:** Dutch HR-GNR definition

Organism	ESBL	CAR	QUI	AMG	CFT	PIP	COT
Enterobacteriaceae	A	A	B	B	-	-	-
*Acinetobacter species*	-	A	B	B	-	-	-
*Stenotrophomonas maltophilia*	-	-	-	-	-	-	A
*Pseudomonas aeruginosa*	-	C	C	C	C	C	-

A: This type of resistance mechanism or resistance against this antimicrobial agent indicates a HR-GNR

B: Resistance against minimal two antimicrobial agents indicates a HR-GNR

C: Resistance against minimal three antimicrobial agents indicates a HR-GNR

*ESBL* extended spectrum beta lactamase, *CAR* carbapenems, *QUI* fluoroquinolones, *AMG* aminoglycosides

*CFT* ceftazidime, *PIP* piperacillin, *COT* co-trimoxazole

Several Dutch studies reported ESBL (colonization) prevalence rates in different human populations and regions ranging from 4.7 to 10.1 % [7–12]. In addition, molecular analyses of ESBL positive isolates found in animals (veal calves, broilers and companion animals) and humans showed several associations that suggest transmission [10–14]. However, exact transmission routes and risk factors are largely unknown since epidemiological links are frequently missing, which limits the interpretation of molecular typing results. Previous studies on livestock-associated methicillin-resistant *Staphylococcus aureus* (LA-MRSA) have shown that differences in prevalence rates exist between different regions of the Netherlands, stressing the importance of regional prevalence studies which may also apply to the HR-GNR (and ESBL) epidemiology [15].

To our knowledge, the above mentioned prevalence studies are the only (recent) studies carried out in the Netherlands that determined the prevalence of ESBL-producing Enterobacteriaceae among different human populations. No studies were found that examined the prevalence and genotypes of all defined HR-GNRs among hospitalized patients (Table 1). Furthermore, the Regional Public Health Laboratory Kennemerland (RPHLK) is a microbiological diagnostic and expertise laboratory that performs infectious disease diagnostics for primary, secondary and tertiary care facilities in the Dutch region Kennemerland allowing the possibility to perform standardized prevalence measurements from a regional point of view.

In this cross-sectional prevalence measurement, we aimed to investigate (1) the HR-GNR prevalence rate, (2) their genotypes and acquired resistance genes, and (3) associated risk factors of HR-GNR colonization among the hospitalized population in the Dutch region Kennemerland.

## Methods

### Ethics statement

According to the Dutch regulation for research with human subjects, neither medical or ethical approval was required to conduct the study since the data were collected as part of each hospitals standard infection control program. Additionally, we received approval to conduct the study from the institutional review board of the Spaarne Gasthuis. The data were anonymized and analyzed under code.

### Study design, setting, participants, data collection and variables

Between 1 October 2013 and 31 March 2014, cross-sectional (point)prevalence measurements were performed in the three regional hospitals in the Dutch region Kennemerland as part of each hospitals infection control program. Rectal swabs were obtained from hospitalized patients (independent of the hospitalization time) at all participating wards (internal medicine, cardiology, neurology, surgery, urology, pulmonology, intensive care unit, pediatrics, geriatrics, orthopedics and gynecology). Outpatients as well as patients on day care were excluded.

Additionally, the following data were obtained from the hospital and laboratory information system in order to identify possible risk factors: (1) basic patient characteristics (gender and age), (2) antibiotic usage (during current admission and up-to six months before admission), (3) admission information (during current admission and up to 1 year before admission) and (4) historic HR-GNR isolates (since January 2008).

### Laboratory detection HR-GNRs

Rectal swabs (Copan eSwab including 1 mL of modified liquid amies) were analyzed for the presence of HR-GNRs at the RPHLK by direct culturing on both an ESBL screening agar (ChromID ESBL-ID, bioMerieux, enriched with a mixture of antibiotics, including cefpodoxime) and a CLED GM20 agar (with 20 mg/L gentamicin, Oxoid). Gram-negative rods growing on these two agars were identified using MALDI-TOF (Bruker Daltonics, Germany). Antibiotic susceptibility testing was performed using the automated system VITEK2 (bioMérieux, France). All isolates suspected for the production of ESBL were confirmed using the combination disk method (ceftazidime and cefotaxime or cefepime with and without clavulanic acid) [16]. Strains suspected for carbapenemase production were confirmed using the modified Hodge test [16]. All positive isolates were stored at −80 °C.

### Molecular characterization of HR-GNR positive isolates by whole genome sequencing

DNA was extracted using the UltraClean microbial DNA isolation kit (Mo Bio Laboratories, Carlsbad, CA, USA) according to the manufacturer’s protocol. A DNA library was prepared using the Nextera XT kit (Illumina, San Diego, CA, USA) according to the manufacturer’s instructions. Subsequently, Whole Genome Sequencing (WGS) was performed using the MiSeq instrument (Illumina) for generating paired-end 250-bp reads, aiming at a coverage of at least 60-fold. *De novo* assembly was performed as described previously using CLC Genomics Workbench v7.0.3 (CLC bio A/S, Aarhus, Denmark) after quality trimming (Qs ≥ 28) with optimal word sizes based on the maximum N50 value [17, 18]. The sequence type (ST) was identified by uploading the assembled genomes to the multilocus sequence type (MLST) server (version 1.7) and the acquired resistance genes were determined with the CGE Resfinder 1.2 tool [19, 20]. STs previously undescribed were submitted to *Stenotrophomonas maltophilia* MLST database (http://pubmlst.org/smaltophilia/), to the *Enterobacter cloacae* MLST database (http://pubmlst.org/ecloacae/) or to the Enterobase database (http://enterobase.warwick.ac.uk/).

### Infection prevention policy in the participating hospitals

The infection prevention policy in the three regional hospitals is based on the Dutch Working party of Infection prevention (WIP), which is considered highly effective and widely accepted by all Dutch hospitals in order to prevent nosocomial spread of Multi Drug Resistant Micro-Organisms (MDROs) [6]. The hospitals infection prevention policy includes the training of hospital staff (such as not wearing hand jewelry and the daily use of clean hospital uniforms). Patient rooms are daily cleaned and disinfected when indicated. As part of the policy, patients with an increased risk of HR-GNR acquisition, such as admission in a hospital abroad are screened before admission and nursed in contact isolation, consisting of: (1) nursing in a single room (2) the use of protective clothing, including gloves and gown and/or mask (when indicated), (3) compliance to hand hygiene protocols and (4) disinfection of medical devices and equipment after use. In addition, an alert pop-up is entered in the hospital information system as warning when HR-GNR positive patients are readmitted to the hospital. In case of an unexpected HR-GNR positive patient, all contacts (patients that were still hospitalized and shared the same room with the index patient for at least 24 h during the current hospital stay) were screened for HR-GNR colonization and isolated (when positive) as described.

### Outcomes and data analysis

The primary outcome was the rectal HR-GNR prevalence rate at patient level. These prevalence rates were calculated by dividing the number of HR-GNR positive patients (and per HR-GNR subgroup) by the total number of sampled patients. Confidence intervals (95 %) of the prevalence rates were calculated using the Wilson score [21]. Proportions (such as prevalence rates and type of micro-organism) were compared between hospitals using the Pearson chi-square test or Fisher’s exact test (when appropriate).

The association between several independent variables (sex, age, historic antibiotic use, antibiotic use during admission, historic admission (up to 1 year), known HR-GNR positive in the past (since January 2008) and time from start admission to sampling) and the dependent variable HR-GNR positivity (yes/no) was examined using logistic regression models. These associations were presented as odds ratio’s, including 95 % confidence intervals and *p*-values. The independent continuous variables were first checked for linearity and when not linear associated reported as quartiles. Univariate significant associations were further analyzed using a multivariate logistic regression model. All analyses were performed using PASW SPSS Statistics version 18.0. Results were interpreted as statistically significant when the *p*-value was < 0.05.

## Results

### Study population

In total, out of 566 eligible patients, 427 patients (75.4 %) were sampled during this present regional prevalence measurement. The mean age (SD) of this study population was 65.1 (21.1) years and 217 patients (50.8 %) were male. The median time (range) between admission and sampling was 3 (0–48) days. From all sampled patients, twelve (2.8 %) patients were known to be HR-GNR positive in the past (since January 2008) of whom three (25.0 %) patient(s) were positive and nine (75.0 %) were negative in the present prevalence measurement. The median time (range) between the first HR-GNR positive isolate and the current prevalence measurement for the three positive and nine negative patients was 414 (69–1991) and 648 (28–2273) days, respectively. A total of 167 (39.3 %) patients used antibiotics during the present hospital stay and 99 (23.3 %) patients used antibiotics up to 6 months before the present hospital stay. A total of 204 patients (48.0 %) were admitted (up to 1 year) before the present hospital stay. Stratified study population characteristics for the separate hospitals are shown in Table 2. From two patients, no demographic and historic data were available and these were excluded from further analyses.

**Table 2:** Demographic characteristics of patients

Patient characteristics	Total^a^	Hospital 1	Hospital 2	Hospital 3^a^
Number of unique patients	427 (100)	126 (29.5)	167 (39.1)	134 (31.4)
Sex				
Male	217 (51.1)	57 (45.2)	90 (53.9)	70 (53.0)
Used antibiotics 6 months before current admission	99 (23.3)	41 (32.5)	34 (20.4)	24 (18.2)
Used antibiotics during current admission	167 (39.3)	33 (26.2)	76 (45.5)	58 (43.9)
Admitted before current admission (up to 1 year)	204 (48.0)	65 (51.6)	77 (46.1)	62 (47.0)
Known HR-GNR positive in the past^b^	12 (2.8)	2 (1.6)	7 (4.2)	3 (2.3)
Mean age in years (SD)	65.1 (21.1)	61.2 (22.9)	65.7 (22.4)	68.1 (16.9)
Median time from admission to sampling in days (Range)	3.0 (0–48)	2 (0–29)	4 (0–48)	4 (0–43)

Data are presented as numbers (%) unless indicated otherwise

Percentages were calculated in reference to the specific hospital

^a^From two patients no demographic characteristics were known (in total: 425 patients with known characteristics and 132 for hospital 3)

^b^Known HR-GNR positive in the past (since January 2008)

### Prevalence of HR-GNRs and micro-organisms in hospitalized patients

A total of 22 patients were culture positive for one or more types of HR-GNRs, resulting in a regional HR-GNR colonization prevalence (95 % CI) of 5.2 % (3.6–7.9). Of these 22 positive patients, 15 were ESBL positive (3.5 % (2.1–5.7)), 7 patients were positive with an isolate resistant towards Q&A (1.6 % (0.8–3.3)) and from one patient (0.2 % (0–1.3)) a *Stenotrophomonas maltophilia* resistant towards co-trimoxazole was isolated. No CPE, multi-resistant *Acinetobacter* species or multi-resistant *Pseudomonas aeruginosa* were isolated. From three patients (0.7 %), more than one distinctive HR-GNR phenotype was isolated of whom one patient (0.2 %) was positive for more than one type of HR-GNR (both an ESBL positive isolate and isolate resistant towards Q&A). Stratified prevalence rates for each hospital and type of HR-GNR are shown in Table 3. No statistically significant differences in prevalence rates were found between hospitals (*p* = 0.180). When patients were divided in two groups based on hospitalization time, a HR-GNR prevalence (95 % CI) of 5.3 % (2.6–10.5) and 4.7 % (2.9–7.9) was found respectively for patients that were 0–1 day hospitalized (7 out of 133 patients) and longer than one day hospitalized (14 out of 292 patients) (*p* = 0.836).

**Table 3:** Prevalence rates of rectal HR-GNRs colonization on a patient level

	Regional		Hospital 1		Hospital 2		Hospital 3	
	Prevalence	95 % CI	Prevalence	95 % CI	Prevalence	95 % CI	Prevalence	95 % CI
HR-GNR	5.2 % (22/427)	3.6 %–7.9 %	2.4 % (3/126)	0.8 %–6.7 %	7.2 % (12/167)	4.6 %–12.8 %	5.2 % (7/134)	2.6 %–10.4 %
ESBL	3.5 % (15/427)	2.1 %–5.7 %	1.6 % (2/126)	0.4 %–5.6 %	5.4 % (9/167)	2.8 %–9.9 %	3.0 % (4/134)	1.2 %–7.4 %
Q&A	1.6 % (7/427)	0.8 %–3.3 %	0.8 % (1/126)	0.1 %–4.3 %	2.4 % (4/167)	0.9 %–6.0 %	1.5 % (2/134)	0.4 %–5.3 %
Other^a^	0.2 % (1/427)	0.0 %–1.3 %	0.0 % (0/126)	0.0 %–0.0 %	0.0 % (0/167)	0.0 %–0.0 %	0.7 % (1/134)	0.1 %–3.4 %

^a^All other HR-GNR beside ESBL and Q&A, see Table 1

Most of the isolated HR-GNR micro-organisms were *Escherichia coli* (73.1 %) followed by *Enterobacter cloacae* (11.5 %), *Klebsiella pneumoniae* (11.5 %) and *Stenotrophomonas maltophilia* (3.8 %). There was no significant difference in the total distribution of all micro-organisms between the three hospitals (*p* = 0.421).

### Molecular characterization of ESBL positive isolates

Twenty-four HR-GNR positive isolates belonging to 22 HR-GNR positive patients were genotyped using WGS. All 16 phenotypically ESBL positive isolates sampled from 15 patients harbored ESBL genes of which one isolate (*Klebsiella pneumonia*, ST48) contained two ESBL genes (*bla*_CTX-M-15_ and *bla*_SHV-11_). ESBL genes found were *bla*_CTX-M-1_ (*n* = 4, 25.0 %), *bla*_CTX-M-15_ (*n* = 3, 18.8 %), *bla*_CTX-M-27_ (*n* = 2, 12.5 %), *bla*_CTX-M-14b_ (*n* = 2, 12.5 %), *bla*_CTX-M-9_ (*n* = 2, 12.5 %), *bla*_CTX-M-14_ (*n* = 1, 6.3 %), *bla*_CTX-M-3_ (*n* = 1, 6.3 %), *bla*_SHV-11_ (*n* = 1, 6.3 %) and *bla*_SHV-12_ (*n* = 1, 6.3 %) (Table 4).

**Table 4:** Molecular characteristics of HR-GNR positive isolates

Patient	Hospital	Species	MLST	HR-GNR type^a, b, c^	ESBL gene(s)	Acquired AMG^d^resistance genes	Acquired QUI^e^resistance genes	AMG^d^resistance^f^	QUI^e^resistance^f^
P1	1	*E. coli*	ST685	ESBL	CTX-M-1	-	-	S	S
P2	1	*K. pneumoniae*	ST48	ESBL	CTX-M-15, SHV-11	strA, strB	oqxA, oqxB, QnrB66	S	S
P3	1	*E. coli*	ST69	Q&A	-	strA, strB, aph(3’)-Ia, aadA5, aac(3)-IId	-	R	R
P4	2	*E. coli*	ST5929^g^	ESBL	CTX-M-1	-	-	S	S
P5	2	*E. coli*	ST69	ESBL	CTX-M-1	aadA5	-	S	S
P6	2	*E. coli*	ST2178	ESBL	CTX-M-3	-	-	S	S
		*E. coli*	ND	ESBL	ND	ND	ND	S	S
		*K. pneumoniae*	ST22	ESBL	CTX-M-15	strA, strB, aph (3’)-Ic, aac(3)-Iia, aac(6’) Ib-cr	oqxA, oqxB, aac(6’) Ib-cr, QnrB66	R	S
P7	2	*E. coli*	ST349	ESBL	CTX-M-14b	strA, strB	-	S	S
P8	2	*E. coli*	ST93	ESBL	CTX-M-14b	-	-	S	R
P9	2	*E. coli*	ST131	ESBL	CTX-M-27	strA, strB, aadA5	-	S	R
P10	2	*E. cloacae*	ST50	ESBL	CTX-M-1	aadA2, aadB	oqxA, oqxB, QnrA1	I	R
P11	2	*E. cloacae*	ST421^g^	ESBL	CTX-M-9	aadB	oqxA, oqxB, QnrA1	S	S
P12	2	*E. cloacae*	ST50	ESBL	CTX-M-9	aadA2, aadB	oqxA, oqxB, QnrA1	I	R
		*E. coli*	ST88	Q&A	-	aph(3’)-Ic	-	R	R
P13	2	*E. coli*	ST69	Q&A	-	strA, strB, aac(3)-IId	-	R	R
P14	2	*E. coli*	ST131	Q&A	-	strA, aac(3)-Iid, aadA5	-	R	R
P15	2	*E. coli*	ST648	Q&A	-	aac(6’) Ib-cr	aac(6’) Ib-cr	R	R
P16	3	*E. coli*	ST10	ESBL	CTX-M-14	strA, strB, aac(3)-IId, aadA1, aadA4, aac(6’) Ib-cr	aac(6’) Ib-cr	R	S
P17	3	*K. pneumoniae*	ST45	ESBL	CTX-M-15	strA, strB, aac(3)-IIa, aac(6’) Ib-cr	oqxA, oqxB, aac(6’) Ib-cr, QnrB66	R	I
P18	3	*E. coli*	ST131	ESBL	CTX-M-27	-	-	S	R
P19	3	*E. coli*	ST635	ESBL	SHV-12	strA, strB, aac(3)-IIb, aacA4, aac(6’) Ib-cr, aac(6’)-IIc	aac(6’) Ib-cr, QnrB4, QnrB66	R	I
P20	3	*E. coli*	ST57	Q&A	-	aph(4)-Ia, aac(3)-IVa, aadA2	-	R	R
		*E. coli*	ND	Q&A	-	ND	ND	R	R
P21	3	*E. coli*	ST131	Q&A	-	strA, strB, aac(3)-Iid, aadA5	-	R	R
P22	3	*S. maltophilia*	ST152^g^	CTX-R	-	aph(3’)-IIc, aacA4, aac(6’) Ib-cr	aac(6’) Ib-cr	ND	ND

^a^: ESBL: Extended Spectrum Beta Lactamase; ^b^: Q&A: combined resistance towards Fluoroquinolones and Aminoglycosides; ^c^: CTX-R: resistance towards Co-trimoxazole; ^d^: QUI: Fluoroquinolones; ^e^: AMG: Aminoglycosides; ^f^: resistance based on VITEK2 results; *S* sensitive, *I* intermediate, *R* resistant, *ND* not determined, *NA* not available, ^*g*^ new sequence type

Eleven of the 16 ESBL positive isolates were determined as *Escherichia coli* (68.8 %). ST131 was found twice (12.5 %) and both isolates harbored the *bla*_CTX-M-27_ gene. All other ESBL positive *Escherichia coli* sequence types were found once (*n* = 1, 6.3 %) and included ST10, ST69, ST88, ST93, ST349, ST635, ST685, ST2178 and a new sequence type (ST5929). Three of the 16 ESBL positive isolates were determined as *Klebsiella pneumonia* (18.8 %) consisting of ST22, ST45 and ST48. Additionally, all three ESBL positive *Klebsiella pneumonia* isolates harbored the *bla*_CTX-M-15_ gene. The other three ESBL positive isolates were determined as *Enterobacter cloacae* (18.8 %) of which ST50 was found twice and a new sequence type (ST421) once. Two of the three ESBL positive *Enterobacter cloacae* isolates harbored the *bla*_CTX-M-9_ gene. In addition, Table 4 also shows phenotypic resistance patterns and identified acquired resistance genes towards Q&A, revealing that the presence of acquired resistance genes in an isolate and phenotypic resistance do not always co-occur.

### Molecular characterization of non-ESBL HR-GNR positive isolates

Eight (33.3 %) of the 24 genotyped HR-GNR positive isolates (belonging to eight patients) were non-ESBL isolates. Seven of these eight isolates showed combined resistance towards Q&A. All seven isolates resistant towards Q&A were determined as *Escherichia coli* of which ST69 and ST131 were both found twice (28.6 %). All other sequence types (ST57, ST88, and ST648) were recovered once (14.3 %). Only one isolate resistant towards Q&A (ST648) harbored the acquired *aac(6’) Ib-cr* gene encoding for resistance towards both Fluoroquinolones and Aminoglycosides. The other isolates resistant towards Q&A harbored only acquired resistance genes encoding for Aminoglycoside resistance although they also showed phenotypic resistance towards Fluoroquinolones. These acquired Aminoglycoside resistance genes were *strA* (*n* = 4, 57.1 %), *strB* (*n* = 3, 42.9 %), *aac(3) -IId* (*n* = 3, 42.9 %), *aadA5* (*n* = 3, 42.9 %), *aph(3’) -Ia* (*n* = 1, 14.3 %), *aph(4) -Ia* (*n* = 1, 14.3 %), *aac(3) -IVa* (*n* = 1, 14.3 %), *aadA2* (*n* = 1, 14.3 %) and *aph(3’)-Ic* (*n* = 1, 14.3 %).

One of the eight non-ESBL HR-GNR positive isolates was determined as *Stenotrophomonas maltophilia*, which showed resistance towards co-trimoxazole (12.5 %) and was characterized as ST152 (new sequence type). This isolate harbored the acquired *sul1* gene encoding for Sulphonamide resistance.

### Associated risk factors for being HR-GNR positive in hospitalized patients

Table 5 shows the associations between independent variables and the dependent variable (HR-GNR positive in the prevalence measurement). Both continuous variables (age and time from admission to sampling) were reported as quartiles (in reference to the first category) since they were not linearly associated with the outcome. Being known HR-GNR positive in the past was the only significant associated risk factor, odds ratio (95 % CI): 7.32 (1.82–29.35), *p*-value = 0.005. The other tested risk factors were associated with an increased risk of HR-GNR positivity, although not significant. The association with being HR-GNR positive in the past did not markedly change after adjustment for possible confounders (antibiotic use (during the current and up to 6 months before the current admission) age, sex, admission before the current admission and time from start admission to sampling) using multivariate logistic regression analysis, odds ratio (95 % CI): 6.54 (1.35–31.61), *p*-value = 0.020 (Table 6). No significant effect modifiers were identified.

**Table 5:** Univariate associations between possible risk factors in clinical patients and being HR-GNR positive

Risk factor	HR-GNR positive patients (*n* = 21)^a^	HR-GNR negative patients (*n* = 404)^a^	Odds ratio (95 % CI)	*P*-value
Sex				
Female	9 (42.9)	199 (49.3)	1 (ref)	-
Male	12 (57.1)	205 (50.7)	1.29 (0.53–3.14)	0.568
Used antibiotics 6 months before current admission	6 (28.6)	93 (23.0)	1.34 (0.51–3.55)	0.559
Used antibiotics during admission	10 (47.6)	157 (38.9)	1.43 (0.59–3.45)	0.425
Admitted before current admission (up to 1 year)	14 (66.7)	190 (47.0)	2.25 (0.89–5.70)	0.086
Known HR-GNR positive in the past	3 (14.3)	9 (2.2)	7.32 (1.82–29.35)	0.005
Age (years)				
Group 1 (0–56 years)	4 (19.0)	103 (25.5)	1 (ref)	-
Group 2 (57–70 years)	5 (23.8)	99 (24.5)	1.30 (0.34–4.98)	0.701
Group 3 (71–79 years)	9 (42.9)	101 (25.0)	2.30 (0.69–7.69)	0.178
Group 4 (80–94 years)	3 (14.3)	101 (25.0)	0.77 (0.17–3.50)	0.730
Time from admission to sampling (days)				
Group 1 (0–1 days)	7 (33.3)	126 (31.2)	1 (ref)	-
Group 2 (2–3 days)	3 (14.3)	90 (22.3)	0.60 (0.15–2.38)	0.468
Group 3 (4–6 days)	3 (14.3)	85 (21.0)	0.64 (0.16–2.53)	0.519
Group 4 (7–48 days)	8 (38.1)	103 (25.5)	1.40 (0.49–3.98)	0.531

^a^In total, data for 425 patients were available for analyses, as for two patients demographic data were unknown

**Table 6:** Multivariate logistic regression model between possible risk factors in clinical patients

	Unadjusted association		Fully adjusted model^a^	
Risk factor	Odds ratio (95 % CI)	*P*-value	Odds ratio (95 % CI)	*P*-value
Sex	1.29 (0.53–3.14)	0.568	1.12 (0.43–2.91)	0.823
Used antibiotics 6 months before current admission	1.34 (0.51–3.55)	0.559	0.84 (0.28–2.51)	0.756
Used antibiotics during admission	1.43 (0.59–3.45)	0.425	1.15 (0.41–3.23)	0.791
Admitted before current admission (up to 1 year)	2.25 (0.89–5.70)	0.086	2.21 (0.80–6.10)	0.126
Known HR-GNR positive in the past	7.32 (1.82–29.35)	0.005	6.54 (1.35–31.61)	0.020
Age (years)				
Group 1 (0–56 years)	1 (ref)	-	1 (ref)	-
Group 2 (57–70 years)	1.30 (0.34–4.98)	0.701	0.84 (0.20–3.45)	0.806
Group 3 (71–79 years)	2.30 (0.69–7.69)	0.178	1.73 (0.49–6.15)	0.395
Group 4 (80–94 years)	0.77 (0.17–3.50)	0.730	0.60 (0.12–2.93)	0.530
Time from admission to sampling (days)				
Group 1 (0–1 days)	1 (ref)	-	1 (ref)	-
Group 2 (2–3 days)	0.60 (0.15–2.38)	0.468	0.47 (0.11–2.01)	0.311
Group 3 (4–6 days)	0.64 (0.16–2.53)	0.519	0.52 (0.12–2.29)	0.385
Group 4 (7–48 days)	1.40 (0.49–3.98)	0.531	1.10 (0.34–3.62)	0.870

^a^Corrected for (1) Antibiotic use during current admission, (2) Antibiotic use up 6 months before current admission, (3) sex, (4) age, (5) admitted before current admission, (6) time from start admission to sampling and (7) known HR-GNR positive in the past

## Discussion

The present study shows the results of a cross-sectional HR-GNR (including ESBL) prevalence measurement in hospitalized patients of three hospitals in the Dutch region Kennemerland (with 650,000 inhabitants). In total, 427 rectal swabs derived from unique patients in these hospitals were analyzed and resulted in a total HR-GNR and ESBL prevalence rate of 5.2 and 3.5 %, respectively. Furthermore, 7 patients (1.6 %) were positive with an isolate resistant towards Q&A, and from one patient (0.2 %) a *Stenotrophomonas maltophilia* resistant towards co-trimoxazole was isolated. In line with other Dutch prevalence studies, no CPE positive bacteria were found, indicating a relatively low prevalence in our region.

Several Dutch studies have previously reported about the ESBL prevalence rates among different human populations. First, Overdevest et al. found a rectal ESBL colonization prevalence of 4.9 % within hospitalized patients [7]. Second, a prevalence survey among 125 residents living in five nursing homes and two (hospital) rehabilitation wards in the central region of the Netherlands showed an ESBL prevalence of 6.0 % [8]. Third, a study among 720 patients with gastrointestinal complaints visiting the general practitioner showed an ESBL prevalence of 10.1 % [9]. Fourth, two studies reported on the ESBL prevalence among travelers (before travel) resulting in an ESBL prevalence rate of 8.6 and 9.0 % [22, 23]. Fifth, a cross-sectional ESBL prevalence measurement performed in a representative sample of the Dutch community population showed an ESBL prevalence of 4.7 % [24]. A comparison between the ESBL prevalence rate in our present study (3.5 %) and the other studies showed that the ESBL prevalence rate in our region was relatively low. A possible explanation for this difference could be the different culture methods that were used. Some studies used an (selective or non-selective) enrichment broth, which is associated with higher sensitivities compared with direct culture methods [25, 26]. Furthermore, differences in population characteristics or a geographical variation in risk factors may also explain the differences in ESBL prevalence rates.

All phenotypic characterized ESBL positive isolates in our study harbored ESBL genes, mostly *bla*_CTX-M_ (88.2 %). WGS showed that the *bla*_CTX-M-1_ (25.0 %), *bla*_CTX-M-15_ (18.8 %), *bla*_CTX-M-14b_ and *bla*_CTX-M-9_ (both 12.5 %) ESBL genes were found most often. In line with our results, another study among hospitalized patients isolated the *bla*_CTX-M-1_ (45.8 %) ESBL gene most often, followed by *bla*_CTX-M-15_ (16.7 %) and *bla*_TEM-52_ (12.5 %) [7]. Surprisingly, a study among a representative sample of the Dutch community population also isolated the ESBL gene *bla*_CTX-M-1_ (35 %), *bla*_CTX-M-15_ (33 %) and *bla*_CTX-M-14_ (18 %) most often, showing that isolates obtained from hospitalized and non-hospitalized individuals share similar ESBL genes [24]. This finding suggests that the positive HR-GNR patients may have already been positive at admission and act as a reservoir for other patients. In addition, studies among cats, dogs and chicken (retail) meat isolated the *bla*_CTX-M-1_ most frequently, indicating that shared reservoirs and/or transmission dynamics exist [11, 12]. Some studies showed other distributions in ESBL genes. A study among symptomatic general practitioner patients with gastrointestinal complaints, found predominantly *bla*_CTX-M-15_ (47 %) ESBL-genes [9]. Furthermore, the two studies that investigated the ESBL prevalence among healthy travelers (before travel) found predominantly *bla*_CTX-M-15_ (47 %) and *bla*_CTX-M-9_ (90 %) ESBL genes [22, 23]. These differences may be explained by heterogeneity in study populations. Travelers, symptomatic general practitioner patients and hospitalized patients possibly show different risk behavior, which may be reflected in the molecular typing results. More research is needed to further elucidate these population differences. As shown, most Dutch ESBL prevalence studies report predominantly *bla*_CTX-M_ ESBL genes and a great variation in *Escherichia coli* sequence types that is in line with our results.

As shown in our study, 6 out of 7 phenotypically characterized isolates resistant towards Q&A had no acquired resistance genes encoding for quinolone resistance, suggesting that quinolone resistance in these isolates was mainly caused by chromosomal mutations. The same was seen for ESBL positive isolates that showed phenotypical resistance towards fluoroquinolones or aminoglycosides (not both). As described earlier by Guan et al.*,* plasmid-encoded quinolone resistance genes do not confer quinolone resistance by themselves, but augment the effect of other resistance mutations [27]. Probably the same conclusion is applicable to acquired Aminoglycoside resistance genes and phenotypic expression. More research is needed to elucidate the role of these resistance genes and phenotypic expression.

Traveling (predominantly to South Asia) and having a high degree of contact with broilers are today’s most important identified risk factors for colonization with ESBL positive bacteria [12, 22, 23, 28, 29]. In our study, we analyzed several potential risk factors for HR-GNR colonization. Antibiotic use (during and/or 6 months before admission) did show a higher odds ratio for HR-GNR positivity (including ESBL), although this association was not significant. The same was found for sex (higher odds for males), ‘admission before the current admission (up to one year)’ and age (for the first three quartiles). Being known HR-GNR positive in the past was the only significantly associated risk factor, also when corrected for possible confounders. Additionally, studies carried out among long term rehabilitation patients also identified ‘being known HR-GNR positive’ as independent risk factor for HR-GNR colonization [30, 31]. This finding suggests that colonization with HR-GNRs persists for a longer period and should not be ignored at re-admission at the hospital. Additional longitudinal studies are needed to quantify the influence of the period of HR-GNR colonization since this information is essential to assess the importance of isolating patients at readmission [6]. Another studied risk factor, time to sampling (from start admission to sampling) was not significantly associated with HR-GNR colonization, indicating that the role of nosocomial colonization was minimal. This finding suggests that basic hygiene in the studied hospitals is good. Even when we excluded the first quartile (0–1 day), as one may hypothesize that HR-GNR positive patients in this group were already positive at admission, no significant association was detected. To elucidate the influence of this possible risk factor, prospective study designs are needed in which all patients are screened at admission and during hospitalization (at specific time intervals).

Furthermore, we suggest that future studies aiming at identifying transmissions routes and/or reservoirs for HR-GNR involving pigs, horses or other (companion) animals, should include both humans and animals (and their isolates) that are epidemiologically linked. When such studies are performed in a longitudinal design, transmission dynamics as well as origin and transmission of resistance genes can be studied (from humans to animals or the other way around).

To our knowledge, no studies are available that determined the colonization prevalence of Q&A resistant Enterobacteriaceae. We recommend for future studies to incorporate all defined HR-GNRs, in addition to ESBLs, in prevalence studies, in order to obtain a more comprehensive overview of colonization with HR-GNRs.

The present study has several limitations. First, only a small number of potential risk factors were included in the risk factor analysis lacking the ability to identify more ‘potential’ risk factors such as contact with HR-GNR positive household members, travel history, food preferences, other medication use, having pets and stay or transfer from a nursing home. Second, as mentioned before, no enrichment broth was used which could have underestimated the HR-GNR prevalence rate. Third, historic data on antibiotic use were retrospectively retrieved from the hospital pharmacy database, which only contains data on clinical prescribed antibiotics, and not on antibiotic use in the primary care setting. Fourth, our study was performed in a single region of the Netherlands with a relatively small number of cases limiting the power to identify all important risk factors. Therefore, our results should be interpreted with caution.

## Conclusion

In conclusion, no local differences in HR-GNR prevalence rates and micro-organisms were found between the three regional hospitals. In addition, the Dutch region Kennemerland showed similar ESBL prevalence rates among the hospitalized patients population in comparison to other Dutch regions. When previously HR-GNR positive patients are readmitted they should be screened for HR-GNR colonization since colonization with GR-GNRs could be prolonged. Molecular typing results showed that comparable ESBL genotypes were found as earlier described in both humans and animals supporting the hypothesis of multiple reservoirs and risk factors. Future studies must focus more on these postulated reservoirs and risk factors with appropriate study designs.
